# Analysis of Muscular Electrical Activity and Blood Perfusion of Upper Extremity in Patients with Hemiplegic Shoulder Pain: A Pilot Study

**DOI:** 10.1155/2022/5253527

**Published:** 2022-09-27

**Authors:** Minghong Sui, Naifu Jiang, Luhui Yan, Chenxi Zhang, Jiaqing Liu, Tiebin Yan, Guanglin Li

**Affiliations:** ^1^Department of Rehabilitation Medicine, Huazhong University of Science and Technology Union Shenzhen Hospital (Shenzhen Nanshan People's Hospital), Shenzhen 518052, China; ^2^CAS Key Laboratory of Human-Machine Intelligence-Synergy Systems, Shenzhen Institute of Advanced Technology (SIAT), Chinese Academy of Sciences (CAS), and the SIAT Branch, Shenzhen Institute of Artificial Intelligence and Robotics for Society, Shenzhen 518055, China; ^3^Guangdong-HongKong-Macao Joint Laboratory of Human-Machine Intelligence-Synergy Systems, Shenzhen 518055, China; ^4^Department of Rehabilitation Medicine, Sun Yat-sen Memorial Hospital, Sun Yat-sen University, Guangzhou 510120, China

## Abstract

**Background:**

Hemiplegic shoulder pain (HSP) is a common symptom for post-stroke patients, which has a severely adverse impact on their rehabilitation outcomes. However, the cause of HSP has not been clearly identified due to its complicated multifactorial etiologies. As possible causes of HSP, the abnormality of both muscular electrical activity and blood perfusion remains lack of investigations.

**Objective:**

This study aimed to analyze the alteration of muscular electrical activity and blood perfusion of upper extremity in patients with HSP by using surface electromyography (sEMG) and laser speckle contrast imaging (LSCI) measurement techniques, which may provide some insight into the etiology of HSP.

**Methods:**

In this observational and cross-sectional study, three groups of participants were recruited. They were hemiplegic patients with shoulder pain (HSP group), hemiplegic patients without shoulder pain (HNSP group), and healthy participants (Healthy group). The sEMG data and blood perfusion data were collected from all the subjects and used to compute three different physiological measures, the root-mean-square (RMS) and median-frequency (MDF) parameters of sEMG recordings, and the perfusion unit (PU) parameter of blood perfusion imaging.

**Results:**

The RMS parameter of sEMG showed significant difference (*p* < 0.05) in the affected side between HSP, HNSP, and Healthy groups. The MDF parameter of sEMG and PU parameter of blood perfusion showed no significant difference in both sides among the three groups (*p* > 0.05). The RMS parameter of sEMG showed a statistically significant correlation with the pain intensity (*r* = -0.691, *p* =0.012).

**Conclusion:**

This study indicated that the muscular electrical activity of upper extremity had a correlation with the presence of HSP, and the blood perfusion seemed to be no such correlation. The findings of the study suggested an alternative way to explore the mechanism and treatment of HSP.

## 1. Introduction

Hemiplegic shoulder pain (HSP) is a common symptom for post-stroked patients [1]. Almost up to 70% of post-stroked patients suffer from HSP, which can have an adverse impact on their rehabilitation outcomes [2]. In order to apply the appropriate therapeutic techniques for HSP, it would be necessary and essential to know the causes of HSP. Nonetheless, the causes of HSP have not been clearly identified due to the complicated multifactorial etiology [3]. As a possible cause, the abnormality of muscle contractions has been investigated in a number of previous studies [4–7].

The stroke-induced weakness, spasticity, and sensory impairment of shoulder muscles are regarded as the relevant factors for shoulder pain [4]. These factors are usually measured via subjective scale [5–12]. Modified Ashworth scale (MAS) and tone assessment scale (TAS) are commonly used to evaluate the muscle tone (spasticity) for hemiplegic patients, while manual muscle testing (MMT) is a highly reliable method for assessing muscle strength [5–7]. Besides, the Fugl-Meyer Assessment for upper extremity (FMA-UE), motor evaluation scale for upper extremity in stroke (MESUPES), and reaching performance scale (RPS) are often applied to assess the upper extremity function in hemiplegic patients [8, 9]. In addition, there are also some objective measures for HSP. For example, the goniometer is used to measure range of motion (ROM; flexion, abduction, internal and external rotation) and the hand grip/held dynamometry and fixed force gauge are used to measure the muscle force [10–12]. However, these methods could not directly assess the activities of individual muscles, which would limit the understanding of the muscle functions in patients with HSP. Surface electromyography (sEMG) provides an objective tool that can be used to assess the activities of individual muscles by measuring the muscular electrical activities. By analyzing sEMG data from relevant muscles related to different types of pains, the strength and endurance of individual muscle contractions would be evaluated, which can provide more detailed electrophysiological information under the mechanism of the neuromuscular etiology of many clinical pains such as low back pain, neck pain, and patellofemoral pain [13–16]. Therefore, it should be also a good way to analyze the mechanism of HSP by using sEMG signals.

On the other hand, the arterial, venous, and lymphatic circulatory pumps of the affected upper extremity require to be activated to facilitate adequate blood flow [17]. Failure of any one of these pumps can lead to the development of regional pain syndrome. The immobility of the hemiplegic shoulder may enhance its development [18]. Consequently, the abnormal alteration of local blood flow may account for the reason of HSP. The laser speckle contrast imaging (LSCI) is a fast, full-field, cheap, and relatively simple imaging method [19]. It can give 2-dimension blood perfusion maps of large surfaces. Compared with other measurement techniques for blood flow such as functional near-infrared spectroscopy (fNIRS) and functional magnetic resonance imaging (fMRI), the LSCI is simpler to operate and more robust [19]. The duration of its preparation and data collection is short. Some studies showed the blood flow/perfusion around targeted muscles could be measured by using LSCI, so that the muscle microcirculation could be analyzed [20, 21]. The LSCI also showed some good findings for assessment of pain-related blood perfusion [22, 23]. Thus, the LSCI would be an additional way to reveal the mechanism of HSP.

It is well known that the abnormal contractions of shoulder muscles in patients with HSP are often observed, which might be produced by the changes of both electrophysiology and blood perfusion. Nonetheless, currently, there is lack of investigation on these two physiological responses when contracting muscles in HSP. Thus, in this study, we aimed to investigate the relationship between the HSP and the two physiological responses (electrophysiology and blood perfusion) by using sEMG recordings and LSCI imaging. For comparison purpose, the hemiplegic patients without shoulder pain (HNSP group) and healthy participants (Healthy group) were also involved in this study. The findings of this study would be helpful to further understand the etiology of HSP and to improve the effectiveness of treatment for patients with HSP.

## 2. Materials and Methods

### 2.1. Experimental Design

In this observational and cross-sectional pilot study, three groups of participants, hemiplegic patients with shoulder pain (HSP), hemiplegic patients without shoulder pain (HNSP), and healthy subjects (Healthy), were recruited. The clinical characteristics and physiological characteristics of each participant were recorded once by an investigator. Five commonly used clinical characteristics including scores of Fugl-Meyer Assessment for Upper Extremity (FMA-UE), Manual Muscle Testing (MMT), Modified Ashworth Scale (MAS), Range of Motion (ROM) for shoulder flexion and abduction, and Visual Analogue Scale (VAS) for shoulder pain intensity were collected from the affected upper extremity in hemiplegic patients. The physiological responses of both sEMG and blood perfusion were recorded from both affected and non-affected upper extremities in the HSP and HNSP groups and both left and right upper extremities in the Healthy group. The experimental procedure was performed in accordance with the Declaration of Helsinki and was approved by the Institutional Ethics Committee (IRB number: 032502). This study was also registered with the Chinese Clinical Trial Registry (ChiCTR2000029051).

### 2.2. Participants

The calculation of sample size in this study was based on data from primary measures (sEMG and blood perfusion) instead of VAS. Unlike VAS for which there are abundant studies, there are few studies on sEMG and blood perfusion in the area of hemiplegic shoulder pain. As such, we have to conduct a trial at first to estimate the appropriate sample size. Through the trial, the measures showed a large difference among Healthy group and two patient groups, which led to a large effect size (8.1458). Generally, a large effect size can result in a small sample size [24]. In our study, we used the G∗Power 3.1.9.2 software to calculate the sample size with the above-mentioned effect size (8.1458), alpha (*α*, 0.05), and power (1-*β*, 0.95), and finally calculated that a sample size of 6 is sufficient for each group. Thirteen hemiplegic patients with shoulder pain and fourteen hemiplegic patients without shoulder pain were recruited from the Huazhong University of Science and Technology Union Shenzhen Hospital (Mar. 2020-Mar. 2021). And thirteen healthy subjects participated in this study as a control group. The same inclusion criteria for both HSP group and HNSP group were aged 18-80 years; first stroked or previous stroked without sequelae; stroked that appeared within one year; limb dysfunction on only one side of the body; stable vital signs; no severe heart, lung, liver, or kidney dysfunction; no coagulation dysfunction. The HSP group was also required to meet the criteria that VAS score of shoulder pain ≥ 4 points. The exclusion criteria for all groups were a history of rotator cuff injury; periarthritis, shoulder surgery, or shoulder trauma; malignant tumor; quadriplegia; severe speech or cognitive dysfunction; mental illness; pain caused by cancer, menopause, or fracture; severe dizziness or a pacemaker. All participants gave their written informed consent before testing.

### 2.3. Physiological Measurements

#### 2.3.1. sEMG Recordings

The shoulder movement is associated with muscles such as biceps brachii, subscapularis, deltoid, pectoralis major, supraspinatus, and infraspinatus [25]. In this study, the sEMG recordings from biceps brachii muscle were selected as a proxy based on the following considerations. (a) Hemiplegic shoulder pain typically appears among stroke patients in the second Brunnstrom stage [2], in which severe muscle spasticity, widely considered the cause of such pain, is often seen in the flexors of these patients [26]. During flexors, the most significant level of spasticity is seen in both biceps brachii and subscapularis, according to previous studies [26] and our clinical observations. Subscapularis is a deep muscle and it is limited to measure its electrophysiological signal by using a surface electrode. Thus in this study, the recordings from biceps brachii were chosen as a proxy. (b) During experiments, we need the patients to do some shoulder movements and maintain the postures for a certain duration for learning electrophysiological mechanism of hemiplegic shoulder pain. For stroke patients with hemiplegic shoulder pain, the flexion and abduction of their shoulder are often difficult to maintain for long enough. On the contrary, the flexion of their elbow is much easier to maintain [26], which mainly controlled by biceps brachii. This is another reason why the recordings from biceps brachii were chosen as a proxy in this study.

**Figure 1:**
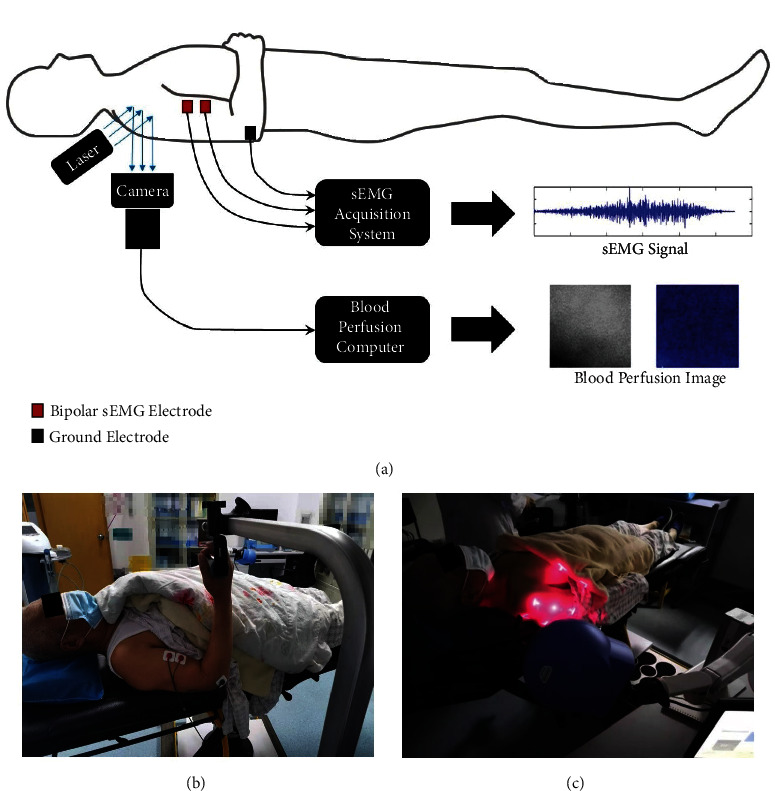
Physiological measurements (sEMG and blood perfusion) of the upper extremity. (a) Design of physiological measurements. (b) Measurement of sEMG in patients. (c) Measurement of blood perfusion in patients.

Then, a pair of bipolar surface electrodes (Ag/AgCl electrode, diameter: 1 cm, inter-electrode distance: 2 cm) were attached to the skin over the biceps brachii. Another one electrode was placed on the bony part of upper extremity as the ground (GND). The placement of the electrodes is shown in Figure 1(a). Before the sEMG electrodes were attached, the skin preparation for sEMG was done according to the following procedures: cleaning the site with alcohol, shaving the electrode site (if the skin surface at the sensor location was covered with noticeable hair), and lightly abrading the skin with fine sandpaper. 8 seconds of sEMG data were recorded when the participant performed a maximum voluntary isometric contraction (MVIC). The MVIC was performed via elbow flexion with the lever arm of an isokinetic dynamometer (Humac2009 system, Human Norm, CA, USA). The participant was asked to repeat the MVIC three times with an interval resting time of 30 seconds to record the sEMG data. By using an EMG acquisition system (Mega ME6000, Mega Electronics, Kuopio, Finland), the sEMG signal was acquired at a sample rate of 1000 Hz.

#### 2.3.2. Blood Perfusion Recordings

Blood perfusion was evaluated in the shoulder area with a PeriCam Perfusion Speckle Imager (PSI) System (Perimed, Stockholm, Sweden) for analysis of complete occlusion after stroke induction (Figure 1(b)). This system provides images using Laser Speckle Contrast Analysis (LASCA) technology, and data on both the dynamics and the spatial distribution of the perfusion throughout the procedure are displayed in real time. The measurement of blood perfusion is based on the speckle pattern of blood cells, which has a relationship with the concentration and mean velocity of the blood cells. The detailed measuring principle of LASCA is displayed in the Supplemental file (available here). A lot of studies have validated the LASCA for showing blood perfusion, by comparing the LASCA with other perfusion measurement tools (e.g., single laser Doppler flowmetry analysis) [27, 28]. Due to the limitation of photon penetration depth, the LASCA technology can only be used to measure the superficial blood perfusion than the deep blood perfusion.

During the procedure, environmental temperature was controlled to approximately 26°C ±1°C and the relative humidity between 50% and 60% whereas the evaluated field was not exposed to direct light. The PSI parameter was set as follows: image acquisition rate, 50 Hz; normal resolution, 0.5 mm; 1 frame per second; 20 ±1 cm of working distance; 5 cm ×5 cm of region of interest (ROI). PIMSoft v1.5.8078 (Perimed, Stockholm, Sweden) was used for recording, saving, and analysis of data. By applying the LASCA technology and using the PIMSoft software, the average perfusion unit (PU) was computed to measure the blood perfusion. It is an arbitrary unit, because it is from the speckle contrast. The higher the PU value, the greater the perfusion observed.

### 2.4. Clinical Measurements

#### 2.4.1. FMA-UE

FMA-UE is a reliable assessment scale to quantitatively evaluate the stroke patients' motor function of upper extremity [29, 30]. It includes 33 items which was divided into 4 subscales: shoulder/elbow (18 items), wrist (5 items), hand (7 items), and coordination/speed (3 items). Each item is scored between 0 and 2 (0 indicates the movement cannot be performed, 1 indicates it is performed partially, and 2 indicates it can be performed fully) with a total score range of 0-66.

#### 2.4.2. MMT

MMT is a clinical procedure for grading the strength of individual muscle or muscle group. The MMT for bicep brachii of affected arm was performed with the patient in the lying position [31]. The MMT score was transformed to 0-12 scale according to the previous study (i.e., 5 score would transform to 12, 5- to 11, 4 to 10, and so forth) [32].

#### 2.4.3. MAS

MAS is a 6-point scale to measure the abnormality in muscle tone. The MAS of bicep brachii of affected arm in all hemiplegic patients was recorded. For data analysis, the 1+ value of MAS was assigned as 2 while 2 was assigned as 3 and so forth [33].

#### 2.4.4. ROM

The ROMs of the shoulder in two different directions (shoulder flexion and shoulder abduction) were measured by a goniometer.

#### 2.4.5. VAS for Shoulder Pain Intensity

VAS is a subjective measure of pain intensity. The range of VAS was from 0 (no pain at that moment) to 10 (worst imaginable pain at that moment).

### 2.5. Data Analysis

Three seconds of stable sEMG data was extracted from original eight seconds of data, in order to remove the movement artifact caused by the paraplegia and pain. Then, a bandpass filter with a range from 10 Hz to 500 Hz and a notch filter of 50 Hz were applied to eliminate the artifact and noise. The root-mean-square (RMS) and median-frequency (MDF) during the three-time MVIC-maintaining period were computed and averaged, using a custom script on MATLAB 2016b (The MathWorks Inc., USA).

RMS is used to measure the amplitude of EMG. It is computed as follows:

(1) $$\begin{array}{c} \text{RMS}=\sqrt{\frac{1}{N}\overset{N}{\underset{k=1}{\sum }}x_{k}^{2}}\;k=1,2,\cdots ,N, \end{array}$$

where *x*_*k*_ is the *k*th sampled sEMG data point; *N* is the sampling number of data points.

MDF is a frequency at which the EMG power spectrum is divided into two regions with equal amplitude. The definition of MDF of sEMG data is given by:

(2) $$\begin{array}{c} MDF=\overset{MDF}{\underset{j=1}{\sum }}P_{j}=\frac{1}{2}\overset{M}{\underset{j=1}{\sum }}P_{j}, \end{array}$$

where *P*_*j*_ is the EMG power spectrum at the frequency bin *j*; *M* is the length of frequency bin.

### 2.6. Statistical Analysis

SPSS 19.0 (IBM, Armonk, NY, USA) was applied to conduct all statistical analyses. The normality of the data set was assessed with the Shapiro–Wilk test. The demographic variables and clinical characteristics variables were compared with *χ*^2^ test and one-way analysis of variance (ANOVA). The RMS value and MDF value from sEMG data and the PU value for blood perfusion were all compared between left and right side in healthy subjects, by using paired *T* test. The difference of these physiological variables among Healthy group, HSP group, and HNSP group was explored using a one-way ANOVA test. Because the homogeneity of variance was violated (Levene's test), this one-way ANOVA test was applied with the Brown-Forsythe correction. The post-hoc analysis was carried out using the Games-Howell test. Because the affected side of stroke patients can be either side, it will produce a bias when managing any side of the healthy subject for the comparison analysis. Thus, we used the average physiological variables of the left and right side of the healthy subjects for analysis. It can help reduce the bias. Pearson's correlation coefficients were used to determine the linear correlation between pain score (VAS) and physiological measurements (sEMG parameters and blood perfusion parameters). Two-tailed *p* values were set at 0.05.

## 3. Results

### 3.1. Demographic and Clinical Baseline Characteristics

We recruited 13 healthy subjects (Healthy group), 13 hemiplegic patients with shoulder pain (HSP group), and 14 hemiplegic patients without shoulder pain (HNSP group) in this study. Most of demographic and clinical baseline characteristics among groups showed no significant difference. Only the ROM of flexion and ROM of abduction showed significant difference between HSP and HNSP groups. The bias caused by the inconsistency of dominant side can be avoided because all subjects were right-handed and the hemiplegic side between the HSP and HNSP groups did not show a significant difference using the *χ*^2^ test. The details are displayed in Table 1.

### 3.2. Comparison of Physiological Responses between Left and Right Side in Healthy Group

**Figure 2:**
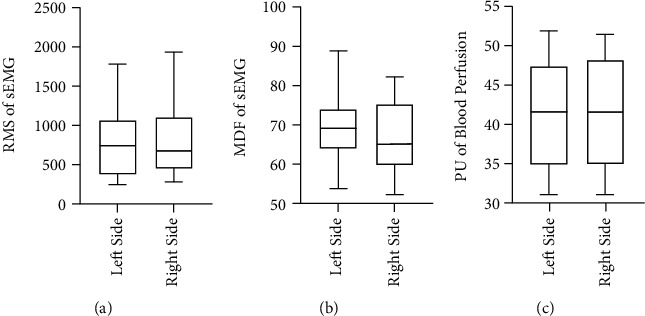
Comparison of sEMG parameters and PU value between left side and right side in the healthy subjects. (a) RMS parameter of sEMG. (b) MDF parameter of sEMG. (c) PU value of blood perfusion.

By using a paired *T* test, the RMS and MDF values from sEMG data showed no significant difference between left and right side in Healthy group (RMS: *p* =0.297, MDF: *p* =0.215). Similarly, the PU value for blood perfusion showed no significant difference between sides in Healthy group (*p* =0.112). The details are displayed in Figure 2.

### 3.3. Comparison of Physiological Responses between HSP, HNSP, and Healthy Groups

**Figure 3:**
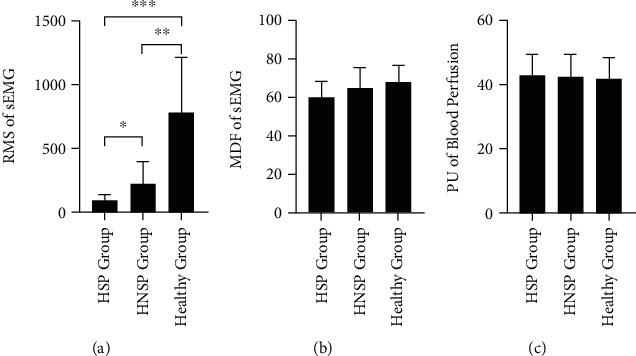
Comparison of sEMG parameters and PU value in the affected side among HSP, HNSP, and Healthy groups. (a) RMS parameter of sEMG. (b) MDF parameter of sEMG. (c) PU value of blood perfusion. ^∗^ means *p* < 0.05. ^∗∗^ means *p* < 0.01. ^∗∗∗^ means *p* < 0.001.

When comparing the physiological responses among the affected side in the HSP group, affected side in the HNSP group, and the left-right-average side in Healthy group, the one-way ANOVA test with Brown-Forsythe correction showed a significant difference of RMS value of sEMG (F(2,15.891) =23.443, *p* < 0.000). The follow-up post-hoc comparison using the Games-Howell test indicated that RMS of sEMG in the HNSP group had significantly higher mean value than that in the HSP group (*p* =0.045), and RMS of sEMG in the Healthy group was significantly higher than that in the HSP group (*p* < 0.001) and HNSP group (*p* =0.002). On the other hand, there was no significant difference on the MDF value of sEMG (F(2,36.020) =2.560, *p* =0.091) and PU value of blood perfusion (F(2,34.800) =0.099, *p* =0.906). The details are displayed in Figure 3.

**Figure 4:**
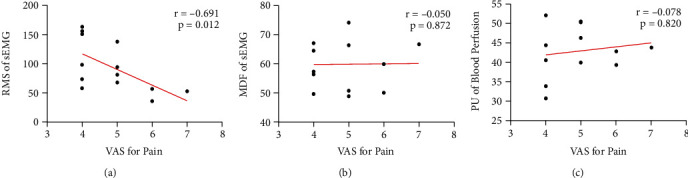
Linear correlation between VAS for shoulder pain intensity and physiological measurements (sEMG parameters and blood perfusion parameters) in the affected side of HSP patients. (a) Correlation between VAS and RMS parameter of sEMG. (b) Correlation between VAS and MDF parameter of sEMG. (c) Correlation between VAS and PU value of blood perfusion.

After the Pearson correlation analysis between the pain score (VAS) and physiological measurements (sEMG parameters and blood perfusion parameters), only the RMS value of sEMG showed a statistically significant correlation with the VAS of pain intensity (*r* = -0.691, *p* =0.012). The details are displayed in Figure 4.

**Figure 5:**
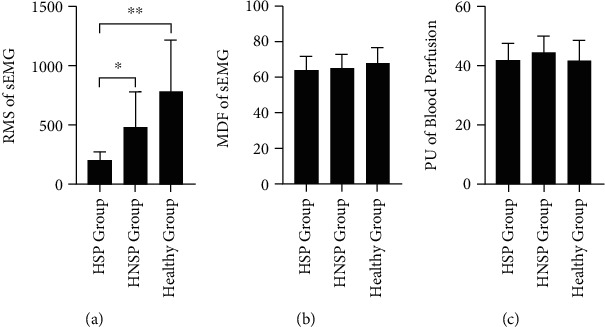
Comparison of sEMG parameters and PU value in the non-affected side among HSP, HNSP, and Healthy groups. (a) RMS parameter of sEMG. (b) MDF parameter of sEMG. (c) PU value of blood perfusion. ^∗^ means *p* < 0.05. ^∗∗^ means *p* < 0.01.

When comparing the physiological responses among the non-affected side in the HSP group, affected side in the HNSP group, and the left-right-average side in Healthy group, the one-way ANOVA test with Brown-Forsythe correction showed a significant difference of RMS value of sEMG (F(2,22.130) =11.600, *p* < 0.001). The follow-up post-hoc comparison using the Games-Howell test indicated that RMS of sEMG in the HNSP group had significantly higher mean value than that in the HSP group (*p* =0.011), RMS of sEMG in the Healthy group was significantly higher than that in the HSP group (*p* =0.001), but not significantly different with that in the HNSP group (*p* =0.116). On the other hand, there was no significant difference on the MDF value of sEMG (F(2,36.433) =0.859, *p* =0.432) and PU value of blood perfusion (F(2,32.447) =0.883, *p* =0.423). The details are displayed in Figure 5.

**Figure 6:**
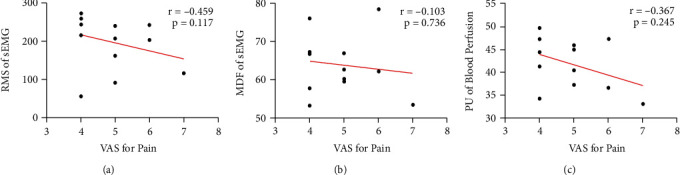
Linear correlation between VAS for shoulder pain intensity and physiological measurements (sEMG parameters and blood perfusion parameters) in the non-affected side of HSP patients. (a) Correlation between VAS and RMS parameter of sEMG. (b) Correlation between VAS and MDF parameter of sEMG. (c) Correlation between VAS and PU value of blood perfusion.

For the Pearson correlation analysis, no significant correlation was shown between pain score and physiological measurements. The details are displayed in Figure 6.

## 4. Discussion

This study adopted sEMG recording and LSCI techniques to measure the muscular electrical activity and blood perfusion of upper extremity in participants with and without HSP. The RMS parameter of sEMG showed significant difference (*p* < 0.05) in the affected side between HSP, HNSP, and Healthy groups. The MDF parameter of sEMG and PU parameter of blood perfusion showed no significant difference in both sides among the three groups (*p* > 0.05).

The RMS parameter from sEMG reflects the intensity of muscular electrical activity [34]. The findings in this study indicate that the single pain symptom has a correlation with the reduction of activities of both affected and non-affected shoulder muscles (HSP vs. HNSP in the affected side: *p* =0.045; HSP vs. HNSP in the non-affected side: *p* =0.011), while the single hemiplegia symptom has a correlation only with the reduction of affected shoulder muscular electrical activity (HNSP vs. Healthy in the affected side: *p* =0.002). The similar findings for the relationship between the hemiplegia and the limbs' muscles can be found in previous studies. Kallenberg, LA et al. found the RMS of motor unit action potential (MUAP) of the affected side is larger and more variable than those of the non-affected side in chronic hemiparetic stroke patients [35]. Chokroverty, S et al. observed the brachial plexus latencies to biceps and deltoid muscles were longer in the affected than in the non-affected sides in some hemiplegic patients [36]. For the relationship between the hemiplegic shoulder pain and the RMS value of bilateral shoulder muscles, there may be three reasons. Firstly, the bilateral disuse muscle atrophy can cause muscle imbalance and potentially cause instability around the shoulder [37]. It might also explain the reason for the pain. Secondly, the cross corticospinal tract from the brain to the muscles may account for this finding. Passing by pyramidal decussation, some of the fibers continue ventrally, forming the corticospinal tract anterior or medial and the remaining crosses to form the corticospinal tract side [38]. The injury or intervention to the hemisphere can also affect the ipsilateral corticospinal tract more or less [39]. Thirdly, the pain sensitization of peripheral/central neural network may affect the control from the spine/brain to the muscles. The pain threshold can decrease with the sensitization in the muscle tissue [40]. It can induce a higher muscle response (amplitude of endplate spikes).

For MDF from sEMG, there is no significant difference among HSP group, HNSP group, and Healthy group in both affected side and non-affected side. Some researchers also found the global MDF of sEMG did not show a significant difference in mean value between the two sides for stroke patients [35, 41]. The MDF generally reflects the recruitment firing rate, which can indirectly assess the muscle fatigue (i.e., muscular endurance) [42]. Fatigue reliably produces a decrease of the frequency feature of sEMG and increase of amplitude feature of sEMG for some specific muscles during static contraction [43, 44]. It indicated the fatigue-related changes in myoelectric properties involved a decrease of conduction velocity (CV) of motor unit action potential (MUAP) [45]. The finding in this study may imply the hemiplegic shoulder pain has no relationship with the alteration of fatigue characteristics of shoulder muscles.

The local superficial blood perfusion in the shoulder, not only in the affected side but also in the non-affected side, did not show any difference among groups. This finding is different with previous ones for hemiplegic limb. Naver, H et al. and Wandklyn, P et al. found both the temperature and blood flow of hand in the hemiplegic side decreased by using the plethysmograph [46, 47]. WC Adams et al. found the blood flow of affected and non-affected feet in the stroke patients was lower than that in the control subjects [48]. They inferred that the reduction of blood flow in hemiplegic limb was likely due to the muscle atrophy. It seemed, in this study, the shoulder pain could increase the superficial blood flow which was decreased by the hemiplegia. Because the LSCI technique can only detect the superficial blood flow [49], it is difficult to conclude whether the local blood flow including superficial and deep blood flow could or could not be affected by the HSP. Further studies using the measurement technique for deep blood flow are required in the future.

There are still several limitations in this study. Firstly, only the sEMG of biceps brachii was collected due to the limited contractions of most shoulder muscles in hemiplegic patients displayed in our previous pilot study. Some advanced techniques such as high-density sEMG and muscle synergy analysis should be used in the future. Secondly, the process of patient recruitment, the proportion of female stroke patients is found to be obviously smaller than that of male patients. In the future, we will try to investigate whether this difference of gender proportion is statistically existed and explore its possible reasons. More patients including female ones will be recruited in further studies in the future. Thirdly, the investigation on the relationship between the periphery neuromuscular system and the central nervous and microcirculation system is missing so the mechanism of HSP cannot be comprehensively explored. This will be investigated in the further study. Fourthly, the shoulder radiography and MRI were not measured in this study. They can be used to assess the degrees of osteoarthritis, rotator cuff tendinits, and tear, which may be the potential causes of hemiplegia shoulder pain [50]. In the further, we will apply these measurement technologies to investigate the mechanism of the hemiplegia shoulder pain. Fifthly, the influence of the duration of hemiplegia on the sEMG as well as the hemiplegic shoulder pain was not investigated. It will be explored in the future study. Finally, the psychological factor was not taken into account in this study. Different psychological state may modulate the pain recording which may cause a bias. We will collect the psychological data (e.g., beck depression inventory questionnaire) in the future.

## 5. Conclusions

By using sEMG and LSCI techniques, it was found the RMS and MDF parameters from sEMG signal in the affected shoulder muscles of HSP group showed significant difference with the Healthy group, while the PU value for blood perfusion showed no significant difference among groups. The muscle imbalance (or muscle dysfunction), caused by the muscle atrophy, impaired motor control, or abnormality of peripheral/central nervous activity, can lead to the instability around the shoulder and the change of sEMG signal. It might explain the reason for the hemiplegic shoulder pain. The findings of the study suggested an alternative way to explore the mechanism and treatment of HSP.

## Figures and Tables

**Table 1:** Participant's baseline characteristics.

	Healthy group (*n* =13)	HSP group (*n* =13)	HNSP group (*n* =14)	*p* value
Male/female (*n*)	10/3	12/1	14/0	0.129∗
Age (years)	54.0 (14.8)	61.9 (10.3)	57.4 (12.2)	0.283^#^
Weight (kg)	67.0 (7.4)	71.3 (13.2)	74.0 (13.5)	0.291^#^
Height (cm)	167.0 (5.5)	169.5 (5.7)	170.0 (5.4)	0.400^#^
BMI (kg/m^2^)	23.9 (1.8)	24.7 (3.5)	25.5 (3.3)	0.373^#^
Right dominant side/total subject number (*n*)	13/13	13/13	14/14	1.000∗
Left/right hemiplegic side (*n*)	N/A	6/7	6/8	0.863∗
Duration of hemiplegia (days)	N/A	91.8 (60.0)	77.4 (52.4)	0.514^#^
FMA-UE	N/A	32.5 (19.4)	38.1 (21.2)	0.486^#^
MMT	N/A	7.2 (3.3)	8.1 (2.2)	0.453^#^
MAS	N/A	0.7 (0.8)	1.1 (1.2)	0.334^#^
ROM for flexion (degree)	N/A	61.2 (50.9)	120.9 (59.3)	0.023^#^
ROM for abduction (degree)	N/A	69.1 (57.6)	120.9 (59.3)	0.030^#^
VAS (0-10)	N/A	4.85 (0.99)	N/A	N/A

HSP means hemiplegic patients with shoulder pain, HNSP means hemiplegic patients without shoulder pain, BMI means body mass index, FMA-UE means Fugl-Meyer assessment for upper extremity, MMT means manual muscle testing, MAS means modified Ashworth scale, ROM means range of motion, VAS means visual analogue scale. ^∗^ means *χ*^2^ test, ^#^ means one-way analysis of variance (ANOVA). N/A means not applicable. Data except male/female (*n*) expressed as mean (standard deviation).

## Data Availability

The data used to support the findings of this study are available from Dr. Minghong Sui (email: meekoo@163.com) upon request.

## Supplementary Materials

Supplementary Materials The detailed measuring principle of Laser Speckle Contrast Analysis (LASCA) is showed in the supplementary material.
