# Supplementary material for: Analysis of Muscular Electrical Activity and Blood Perfusion of Upper Extremity in Patients with Hemiplegic Shoulder Pain: A Pilot Study
Source: Neural Plast. 2022 Sep 27;2022:5253527. doi: 10.1155/2022/5253527 (PMC9532142; doi:10.1155/2022/5253527)
Supplement: Supplementary Materials — The detailed measuring principle of Laser Speckle Contrast Analysis (LASCA) is showed in the supplementary material. [file 5253527.f1.docx]

Measuring Principle of Laser Speckle Contrast Analysis (LASCA)

The blood perfusion is measured using the LASCA technology. LASCA stands for Laser Speckle Contrast Analysis. Illumination of a tissue by coherent, monochromatic laser light produces an interference pattern (speckle pattern) on the tissue surface. This speckle pattern is recorded by a camera inside the PeriCam PSI head, digitized, and transferred to a PC, where the image data is processed and blood perfusion images are generated.

When the illuminated object is static, the speckle pattern is stationary. When moving particles (such as blood cells) are present in the sampled tissue, the speckle pattern will fluctuate over time. By analyzing these intensity fluctuations, information about the blood perfusion in the tissue can be obtained. Areas of high perfusion produce a rapidly changing pattern, and therefore a blurred image with low spatial contrast. Spatial contrast is defined as the standard deviation of the intensity fluctuations divided by their mean intensity, in a small region of the image. The spatial contrast has been found to correlate with blood flow.

By applying the LASCA technology and using the PIMSoft software of PeriCam PSI, the average perfusion unit (PU) was computed to measure the blood perfusion. It is an arbitrary unit, because it is from the speckle contrast. The higher the PU value, the greater the perfusion observed.

Thus, the measurement of blood perfusion is based on the speckle pattern of blood cells, which has a relationship with the concentration and mean velocity of the blood cells.
